# The perception of apathy by caregivers of patients with dementia in Parkinson's disease

**DOI:** 10.1590/s1980-5764-2016dn1004014

**Published:** 2016

**Authors:** Carlos Henrique Ferreira Camargo, Rafael Arthur Serpa, Thiago Matnei, Jivago Szpoganicz Sabatini, Hélio Afonso Ghizoni Teive

**Affiliations:** 1 Universidade Estadual de Ponta Grossa - Hospital Universitário Regional dos Campos Gerais, Ponta Grossa, PR, Brazil.; 2 Universidade Federal do Paraná - Hospital de Clínicas, Serviço de Neurologia, Curitiba, PR, Brazil.

**Keywords:** Parkinson's disease, dementia in Parkinson's disease, apathy, depression

## Abstract

**Background:**

Apathy is one of the main neuropsychiatric symptoms in patients with Parkinson's disease (PD) and is associated with Parkinson's disease dementia (PDD).

**Objective:**

To identify the characteristics of apathy in individuals with PDD according to caregiver perception.

**Methods:**

Thirty-nine patients with PD according to MDS criteria for PDD were included. The following scales were used: the Hoehn and Yahr, the Unified Parkinson's Disease Rating Scale III, Scales for Outcomes in Parkinson's Disease-Cognition (SCOPA Cog), the Montgomery-Åsberg Depression Rating Scale (MADRS) and the Apathy Evaluation Scale (AES).

**Results:**

A total of 97.4% of the patients showed results consistent with apathy. Analysis of question 14 of the AES revealed no correlation with the total result of all the questions [r=–1293, r^2^=0.0167, 95%CI (–0.4274 to 0.1940), P=0.2162], however, there was a correlation of responses to the same question with depression data on the MADRS scale [r=–0.5213, r^2^=0.2718, 95%CI (–0.7186 to –0.2464), P=0.00033].

**Conclusion:**

Apathy is a disorder associated with PDD. However, the scoring scheme of the AES questions can lead to different interpretations of caregiver responses, highlighting limitations of the tool for use in studies of PDD.

## Abstract (pt)

**Embasamento:**

A apatia é uma das principais manifestações neuropsiquiátricas dos pacientes com doença de Parkinson (DP) e está associada com a demência da doença de Parkinson (DDP).

**Objetivo:**

Identificar as características da apatia em indivíduos com DDP através impressão dos cuidadores.

**Metodos:**

Foram avaliados 39 pacientes com DP de acordo com os critérios da MDS para DDP. Foram usadas as escalas de Hoehn e Yahr, *Unified Parkinson's Disease Rating Scale III, Scales for Outcomes in Parkinson's Disease-Cognition* (SCOPA Cog), Escala de Depressão de Montgomery-Asberg (MADSR) e Escala de Avaliação de Apatia (AES).

**Resultados:**

97,4% dos pacientes apresentaram resultados condizentes com apatia. Analisando-se a questão 14 da AES, não foi encontrado correlação com o resultado total de todas as questões [r=–1293, r^2^=0,0167, 95%IC (–0.4274 a 0.1940), P=0,2162], porém, houve uma correlação das respostas da mesma questão com os dados de depressão pela escala MADSR [r=–0.5213, r^2^=0.2718, 95%IC (–0.7186 a –0.2464), P=0.00033].

**Conclusão:**

A apatia é um distúrbio relacionado com a DDP. Entretanto, a forma de pontuação das questões da AES pode induzir a diferentes interpretações das respostas dos cuidadores levando-se a um questionamento sobre a limitação da ferramenta para estudos em DPP.

## INTRODUCTION

Numbering among the most common neuropsychiatric features in Parkinson's disease (PD) are dementia, apathy and depression,^1-4^ sometimes confounded because they are closely related.^5^ Parkinson's disease dementia (PDD) has a prevalence of 30-40% in PD patients and a cumulative prevalence of 48-75% after 15 years of follow-up.^6^ Apathy is a frequent disorder in PD^7,8^ with a prevalence of 16-60% in PD and 50% in PDD.^5,6,9,10^ It can precede the onset of symptoms, tends to regress after the initiation of treatment with dopamine, and returns as PD progresses, with greater frequency in the presence of PDD.^5^ Apathy has been associated with greater executive motor dysfunction and higher risk of developing dementia relative to PD patients without apathy.^11^

Although depression is often confused with apathy, clinical evidence helps differentiate the latter from depression, a relatively common symptom in PD.^2,3,12^ The main differentiating feature is that apathy typically presents with predominantly "neutral" mood, while in depression, mood is clearly negative, causing emotional suffering.^8^ Further evidence is that the occurrence of apathy can be present in the absence of depression and vice-versa.^8,13^ In addition, predominantly apathetic signs and symptoms are evident, such as reduced initiative, decreased participation in external activities, loss of interest in activities of daily living, less interest in starting new activities, emotional indifference, decreased emotional reactivity, lower level of affection than usual, and a lack of concern with the feelings or interests of others.^11^

The objective of the current study was to identify the characteristics of apathy in individuals with PDD according to caregiver perception of patient behavior.

## METHODS

A total of 39 patients with PD were selected according to the diagnostic criteria of the Brain Bank of London for Parkinson's disease^14^ and with PDD according the criteria of the Movement Disorder Society (MDS) for PDD,^15^ seen at the Neurology Service of the Hospital Universitário Regional dos Campos Gerais (HURCG) and at INOVARE Serviços de Saúde Ltda., who agreed to participate in the study. Patients that exhibited clinical conditions which precluded an adequate cognitive assessment and/or application of the proposed tests were excluded:

(a) advanced clinical conditions of the disease;
(b) the presence of psychotic symptoms;
(c) the presence of another dementia other than that associated with PD.

The study was approved by the Research Ethics Committee (COEP) of the Universidade Estadual de Ponta Grossa (reference n^#^ 631.285 FA).

The caregivers were interviewed prior to clinical and cognitive assessment of the patients. Only 2 (27.5%) caregivers were professionals, 18 (47.37%) were patients' sons or daughters, while 18 (47.37%) were patients' spouses. Regarding, education of caregivers, 3 (7.9%) had higher education, 13 (34.21%) had completed high school, and 22 (57.9%) had complete or incomplete primary education.

All patients were evaluated in the *ON* phase of therapy with L-DOPA, preferably at two hours after use of the medication. The clinical evaluation was performed by trained staff in movement disorders. Patients were classified for abnormalities using the Hoehn and Yahr scale^16^ and the Unified Parkinson's Disease Rating Scale III (UPDRS-III).^17^ Cognition was assessed by the test from the Scales for Outcomes in Parkinson's Disease-Cognition (SCOPA-Cog). Out of the maximum 43 points, test scores <22 points indicated cognitive impairment.^18^ Depression was measured using the Montgomery–Åsberg Depression Rating Scale (MADRS). The values considered for analysis were 0-7 for the absence of symptoms and above 8 for presence of depression.^19^ Apathy was quantified using the Brazilian version of the modified Apathy Evaluation Scale (AES)(14 questions –1992) for caregivers.^20^ Values above 14 (possible score 0-72) indicated apathy.^12,13^ The 14 questions can be found in Table 1.

**Table 1:** Apathy evaluation scale.

Original version of the Apathy Scale	Brazilian caregiver version of the Apathy Scale
1. Are you interested in learning new things?	1. Você está interessado em aprender coisas novas?
2. Does anything interest you?	2. Alguma coisa te interessa?
3. Are you concerned about your condition?	3. Você está preocupado com sua condição?
4. Do you put much effort into things?	4. Você se esfoça demais nas coisas que faz?
5. Are you always looking for something to do?	5. Você está sempre procurando alguma coisa para fazer?
6. Do you have plans and goals for the future?	6. Você tem planos para o futuro?
7. Do you have motivation?	7. Você tem motivação?
8. Do you have the energy for daily activities?	8. Você tem energia para atividades diárias?
9. Does someone have to tell you what to do each day?	9. Alguém tem que te falar o que fazer a cada dia?
10. Are you indifferent to things?	10. Você está indiferente para as coisas?
11. Are you unconcerned with many things?	11. Você anda despreocupado com muitas coisas?
12. Do you need a push to get started on things?	12. Você precisa de uma empurrão inicial para começar as coisas?
13. Are you neither happy nor sad, just in between?	13. Você não está nem feliz nem triste, simplestemente um meio termo?
14. Would you consider yourself apathetic?	14. Você se consideraria apático?
**Score:** not at all (3) slightly (2) some (1) a lot (0)	**Pontuações para cara pergunta:** De jeito nenhum (3) Um pouco (2) mais ou menos (1) Muito (0)

Pearson's correlation coefficients were used to determine correlations. Fisher's exact test was employed for differences between found and expected values . The results are expressed as mean±standard deviation or as value followed by the 95% confidence interval (CI) [r (95%)]. Differences were considered significant for P<0.05. The statistical analysis was performed with the *Statistics for Windows* software release 99.

## RESULTS

Among the 39 patients, the male-female ratio was 1.78:^1^. The average age of the patients was 70.7±10.93 years, with high disease duration and time in use of L-DOPA. The motor abnormalities detected by the UPDRS and H&Y scales also showed advanced stages of the disease (Table 2).

**Table 2:** Epidemiological data and values on the UPDRS-III, SCOPA-COG, MADRS and AES for the patients with Parkinson’s disease.

		PDD
N		39 (79.59)
Gender	Male	25 (64.1%)
	Female	14 (35.9%)
Age		70.7±10.93
Age at onset of symptoms		61.11±12.49
Disease duration		8.43±8.99
Time in use of L-DOPA		5.18±5.01
UPDRS-III		22.64±11.41
Hoehn and Yahr		2.23 ±1.28
Educational Level		5.89±4.97
SCOPA-COG		10.82±6.03
MADRS		14.05±9.55
AES		21.51±3.84

PD (Parkinson’s disease); age, educational level, disease duration, disease duration and time in use of L-DOPA in years; UPDRS-III (Unified Parkinson’s Disease Rating Scale); SCOPACog (Scales for Outcomes in Parkinson’s Disease-Cognition). MADRS (Montgomery-Åsberg Depression Rating Scale); AES (Apathy Evaluation Scale).

Only one patient with PDD did not exhibit apathy. This patient also had no depressive symptoms. Of the 38 patients with apathy, 27 (71.05%) had some degree of depression and 11 (28.95%) had no symptoms sufficient to characterize depression. There was a weak inverse correlation between the values of the MADRS and AES in patients with PDD [r=–0.2722; r^2^=0.0741; 95%CI (–0.0473 to 0.5412), *P*=0.0468].

**Figure 1:**
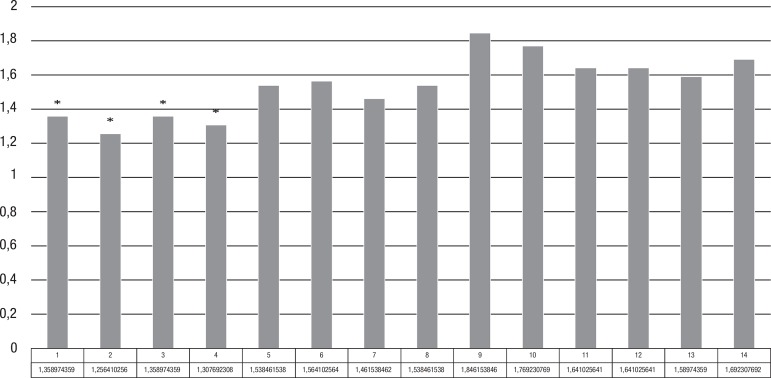
Responses of caregivers to the 14 questions of the AES.

During the implementation of the instrument chosen to measure apathy, a difference in scoring scheme between Questions 1-8 and Questions 9-14 was noted. While in the first group of questions the higher the score achieved, the greater the incapacity of the patient, in the second group, the higher the score, the lower the incapacity evaluated. Question 9, on mood, planning capacity and execution and interest, had the highest response scores (1.846±1.026). Thus, for the question "Does anybody have to tell him/her what he/she needs to do each day?" The response indicates a negative effect, i.e., a low degree of apathy. Question 2, which deals with interest, had the lowest response values (1.256±0.868). Hence, for the question "Is there anything that interests him/her?" positive answers also indicate a low degree of apathy. For question 14, which directly addresses perceived apathy, scores were highest (1.692±1.089), pointing to a lower perception of apathy in patients with PDD. The values of the responses to question 14 differed from values observed in the first questions (Figure 1).

There was no correlation between the data from the question 14 (Do you consider him/her apathetic?) and the overall results of the scale [r=–0.1293, r^2^=0.0167, 95%CI (–0.4274 to 0.1940), P=0.2162], i.e., the impression of the caregivers about the state of apathy of patients was not consistent with the overall results of the scale for apathy identification. However, there was an inverse correlation between the scores on question 14 and the data for depression as measured by the MADRS scale [r=–0.5213, r^2^=0.2718, 95%CI (–0.7186 to –0.2464), P=0.00033], i.e., the apathy perceived by caregivers was associated with a higher level of depressive symptoms in patients.

## DISCUSSION

Of the 39 patients evaluated, 97.4% had high scores on the AES, confirming apathy as a relevant disorder associated with PDD.^6,7^ The rate found was above those found in the literature of 23-50% in patients with PDD.^6^ This high prevalence of apathy in the study might be explained by the more advanced stages of PD in the patients assessed. At advanced stages, cerebral cortex deficits occur through depletion of cortical dopamine and not by dysfunction of the frontal lobe due to incomplete afferents through depletion of dopamine in the striatum.^6,21^ Apathy, in general, is associated with these changes and with more severe cases of the disease, as observed in patients of the present study, with a high average of duration of disease and abnormalities.^1,5^

An inverse correlation between depression and apathy, and independence between the presence of apathy and depression was found in the PDD patients, corroborating findings of other studies.^12,22^ However, the perception of the state of apathy of patients by caregivers was unreliable with confounding of apathy and depression symptoms. These data draw attention to a possible difficulty of transfer of information from the caregiver to the physician, where this may lead to an erroneous diagnosis and wrong treatment. The distinction of these syndromes is of extreme importance in the therapeutic setting, averting a situation where apathy in patients with PDD may be precipitated or worsened by the inadvertent use of serotonin reuptake inhibitors^5,6,11^

The scale was applied to caregivers with different socioeconomic levels, where the definition of "interests" may have been misinterpreted, leading to an erroneous conclusion about the patient's diagnosis. However, the fact that higher scores for some questions in the scale indicated the presence of apathy yet for others indicated lack of apathy may represent an important bias altering and possibility invalidating the results. This is perhaps the main limitation of studies of apathy in PD patients.^12,13,22^

The present study corroborated the high prevalence of apathy in patients with PDD. However, the limitations of the instrument used for measuring apathy were highlighted, including the lack of uniformity among its items, although the tool has been previously validated for this use. Therefore, although the caregiver responses in this study pointed to the existence of apathy in patients with PDD, these results require further validation in larger groups and using other research tools.
